# AI-Supported Prediction of Vesicoureteral Reflux in Children Based on Cystoscopic Configuration of the Ureteric Orifice

**DOI:** 10.3390/jcm15166246

**Published:** 2026-08-12

**Authors:** Vanessa Wolfschluckner, Tristan Till, Sebastian Tschauner, Georg Singer, Alireza Basharkhah, Holger Till

**Affiliations:** 1 Department of Paediatric and Adolescent Surgery, Medical University of Graz, Auenbruggerplatz 34, 8036 Graz, Austria; vanessa.wolfschluckner@medunigraz.at (V.W.); georg.singer@medunigraz.at (G.S.); alireza.basharkhah@medunigraz.at (A.B.); 2 Division of Paediatric Radiology, Department of Radiology, Medical University of Graz, 8036 Graz, Austria; tristan.till@medunigraz.at (T.T.); sebastian.tschauner@medunigraz.at (S.T.)

**Keywords:** artificial intelligence, machine learning, vesicoureteral reflux, pediatric urology, cystoscopy, ureteric orifice, YOLO, EfficientNet

## Abstract

**Background**: Vesicoureteral reflux (VUR) is a common pediatric urological disorder traditionally diagnosed by voiding cystourethrography (VCUG). Although ureteric orifice (UO) morphology during cystoscopy correlates with VUR severity, its assessment remains subjective. To our knowledge, artificial intelligence (AI) has not previously been applied to cystoscopic images for VUR prediction. We aimed to develop and evaluate the first AI model for predicting VUR from pediatric cystoscopic images and videos. **Methods**: In this retrospective single-center study, cystoscopic videos from children with and without VUR diagnosed by VCUG were analyzed. Individual frames were extracted, anonymized and annotated according to VUR grade. Four YOLOv12 object detection models were trained to classify UOs as no/grade I, low-grade (grades II–III), or high-grade (grades IV–V) VUR. To better understand sources of model failure, additional experiments evaluated binary image classification and class-agnostic UO localization. Finally, frame-level predictions were aggregated across complete cystoscopic sequences using majority voting to assess UO-level performance. **Results**: Three-class object detection demonstrated limited frame-level performance, with a maximum mAP@50 of 0.37 and mAP@50–95 of 0.17. Binary classification achieved a macro precision of 0.62, recall of 0.58, and F1 score of 0.49. Class-agnostic object detection improved localization performance (mAP@50 0.63). Aggregating predictions across complete video sequences substantially improved diagnostic performance, achieving UO-level accuracies of up to 77%, with leave-one-out cross-validation accuracies ranging from 0.69 to 0.77. **Conclusions**: This proof-of-concept study demonstrates the feasibility of AI-assisted VUR prediction from pediatric cystoscopic videos. While frame-level performance was limited, sequence-level aggregation markedly improved diagnostic accuracy, highlighting the importance of temporal information for future video-based AI models in pediatric endourology.

## 1. Introduction

Vesicoureteral reflux (VUR) describes the retrograde flow of urine from the bladder into the upper urinary tract and represents the most common urological malformation in children, affecting approximately 1–2% of the general pediatric population and up to 30–45% of children presenting with a febrile urinary tract infection (UTI) [1]. Left untreated or improperly managed, VUR can result in recurrent pyelonephritis, renal scarring, chronic kidney disease, and ultimately end-stage renal disease in children and young adults [2].

Current diagnostic pathways rely principally on voiding cystourethrography (VCUG), which remains the gold standard for grading VUR according to the International Reflux Study Committee (IRSC) classification (grades I–V) [3]. However, VCUG is invasive, requires urinary catheterization, and exposes patients to ionizing radiation. Moreover, VCUG grading is notoriously operator-dependent: inter-rater agreement among radiologists has been reported at only 59% in well-resourced multi-centre trials [4].

The anatomical appearance of the ureteric orifice (UO) during cystoscopy has long been recognized as a correlate of VUR severity. Based on the embryological Mackie–Stephens ureteral bud theory, ectopically positioned ureteral buds produce orifices that are laterally displaced, tunnel-deficient, and morphologically abnormal [5]. Lyon and colleagues classified UO configurations from normal cone-shaped through stadium, horseshoe, to golf-hole forms, with the latter two correlating with progressive degrees of reflux [6]. A recent clinical series confirmed that UO shape correlates significantly with VUR grade, with horseshoe and golf-hole configurations comprising the majority of severe VUR cases [7]. The dynamic hydrodistention (HD) classification introduced by Kirsch and colleagues provides an additional, more objective endoscopic grading tool with high interobserver concordance [8], though its predictive value for de novo contralateral VUR remains limited [7]. Despite these established morphological correlates, cystoscopic assessment of the UO performed by experienced pediatric urologists remains highly subjective, with significant variability depending on distension state, cystoscope angle, and lighting conditions. These observations suggest that diagnostically relevant information is present within cystoscopic appearance but it is difficult to assess reproducibly. These limitations make cystoscopic assessment an attractive target for AI-assisted image analysis, which may offer greater objectivity and reproducibility than conventional visual interpretation.

Artificial intelligence (AI) and machine learning (ML), particularly convolutional neural networks (CNNs), have demonstrated transformative potential in image-based medical diagnostics. In pediatric urology, AI applications have been expanded to include UTI risk stratification, prediction of VUR spontaneous resolution, VCUG-based grading, and hydronephrosis classification [9,10]. The AI-PEDURO living database of AI applications in pediatric urology identified 17 studies applying AI to VUR or UTI outcomes, with neural networks, tree-based algorithms, and support vector machines being the most commonly employed approaches [9]. Most notably, Li et al. (2024) developed Deep-VCUG, a ResNet-101 ensemble model for automated VCUG grading, achieving AUCs of 0.96 on internal and 0.94 on external multi-institutional testing sets [11], a landmark result demonstrating that AI-assisted VUR grading can match or exceed clinician performance on radiological data.

However, no prior study has applied AI to cystoscopic video data for VUR prediction in children. Translating AI from radiological to endoscopic video data introduces a distinct set of challenges: endoscopic images are susceptible to motion blur, specular reflections, variable lighting, and anatomical obscuration; datasets are smaller and the diagnostic signal is distributed across a temporal sequence rather than contained in a single frame. This study addresses precisely these challenges. We present the development, iterative optimization, and performance evaluation of the first AI model for VUR prediction based on cystoscopic UO morphology, and discuss the methodological lessons learned for future AI development in pediatric endourology.

## 2. Materials and Methods

### 2.1. Ethics and Patient Cohort

Following institutional ethical approval (36-262 ex 23/24), a total of 79 pediatric patients undergoing cystoscopy due to VUR and other urological diseases listed below were included in this retrospective study, comprising 51 boys (64.6%) and 28 girls (35.4%). The mean age at cystoscopy was 4.8 ± 5.0 years (range, 0–17.6 years). Patients with duplex collecting systems, obstructive ureteral orifices, secondary vesicoureteral reflux (e.g., associated with posterior urethral valves) or pronounced ureteral ectopia were excluded from the study.

Patients with other diseases including hypospadias (n = 4), urethral injury (n = 3), ureteropelvic junction obstruction (UPJO; n = 5), posterior urethritis (n = 2), and other less frequent indications (n = 11) were included as controls. In addition, five patients with neurogenic bladder dysfunction and negative VCUG findings were also included as controls. The remaining 49 patients underwent VCUG before cystoscopy as part of the diagnostic evaluation for VUR. None of the patients has undergone deflux or urological surgery before the cystoscopies included in the study.

VUR was diagnosed using VCUG in 41 of the 49 patients, all of whom subsequently underwent cystoscopy. Bilateral VUR was present in 23 patients (56.1%). Overall, 117 UOs were included in the analysis.

On the left side, VUR was absent in 25 ureteral orifices, while grade I VUR was present in 5, grade II in 8, grade III in 10, grade IV in 9, and grade V in 3 ureteral orifices. On the right side, VUR was absent in 34 ureteral orifices, while grade I VUR was present in 4, grade II in 4, grade III in 4, grade IV in 9, and grade V in 2 ureteral orifices.

### 2.2. Image Extraction and Annotation

Individual fully anonymized images were extracted from cystoscopic video recordings and labeled by two experienced pediatric urologists with 5 and 15 years of experience using the Computer Vision Annotation Tool (CVAT), an open-source annotation platform well established in surgical AI development. Annotations classified UOs as representing high-grade VUR (grades IV–V), low-grade VUR (grades II–III) and grade I/no VUR. Therefore, the reference standard was the grade of reflux using the results from the VCUG if available. The control patients who did not receive a VCUG were patients who were asymptomatic and without pathological sonographic results. Table 1 shows the final proportions of UOs by class in our dataset.

We split the dataset into 27,393 images from 91 UO for training and 9211 images from 26 UO for validation. Crucially, all training and validation data splits were conducted strictly at the UO level rather than by random image allocation. Randomly splitting continuous video frames across sets causes severe data leakage, as adjacent frames are near-duplicates.

### 2.3. Model Training and AI Performance Metrics

As described in detail below, three different evaluation tasks were performed on a workstation with one NVIDIA RTX 3060 GPU with 12GB of RAM.

First, we trained and evaluated models on a three-class object detection task, the most natural fit for the nature of the dataset, locating no, low-grade, or high-grade reflux on a per-image basis. Four models of the YOLOv12 family [12] with different sizes and parameter counts (YOLOv12n, YOLOv12s, YOLOv12m, YOLOv12l) were trained for 10 epochs each. The configuration of the models is shown in Table 2.

Otherwise, standard hyperparameters were used. All four YOLOv12 models were trained for a short, fixed budget of 10 epochs using randomly initialized weights. To prevent overfitting on highly correlated consecutive video frames, we implemented several strict countermeasures. First, the data split was maintained exclusively at the orifice-level to ensure that validation metrics would accurately expose any overfitting rather than masking it through adjacent-frame leakage. Second, we applied early stopping based on validation fitness, retaining checkpoints with the highest validation performance. Third, robust regularization was utilized throughout training, including weight decay and data augmentations, to limit the models’ capacity to memorize the dataset.

We report frame-based mean average precision (mAP@50, mAP@50-95) and per-frame accuracy as our primary evaluation metric. Second, we simplified the original task along two axes to identify the reason for the weak performance of the first task. To isolate classification capabilities (i.e., no requirement to localize the UO), additional experiments were performed on a simpler task for binary classification of no-/low- and high-grade reflux, based on the same dataset. For this task, an EfficientNet [13] architecture was employed and we report precision, recall and F1 score as our primary metrics. Additionally, to isolate localization capabilities (i.e., no requirement to classify the UO but only to localize the UO), we created a single-class object detection dataset. For this task, YOLOv12 models were used. We report frame-based mean average precision (mAP@50, mAP@50-95) as our primary evaluation metric.

Third, instead of interpreting accuracy on a frame-by-frame basis, we observed whether sequence-level aggregation yields correct conclusions in cross-sequence patient studies. Results are collected by re-using the YOLOv12 models from task 1. We report majority-voted accuracy on UO-level as our primary metric.

To aggregate frame-level predictions into sequence-level classifications, we implemented a temporal majority voting scheme. For each individual video frame within a sequence of a single ureteric orifice, candidate detections with a class confidence score below a specified threshold were discarded. The frame’s vote was then assigned to the class of the remaining detection that had the highest confidence score. If no detections in a frame exceeded the threshold, that frame was logged as a non-prediction.

The final sequence-level classification for the entire ureteric orifice was defined as the class that received the majority of votes across all predicting frames in the sequence. To prevent threshold-selection bias, we employed a leave-one-out (LOVO) cross-validation strategy: for each validation orifice in turn, the optimal threshold was determined using only the remaining orifices, ensuring the evaluated orifice never contributed to its own threshold selection. The accompanying 95% confidence intervals were computed via bootstrap resampling at the orifice level.

## 3. Results

### 3.1. Three-Class Object Detection

**Figure 1:**
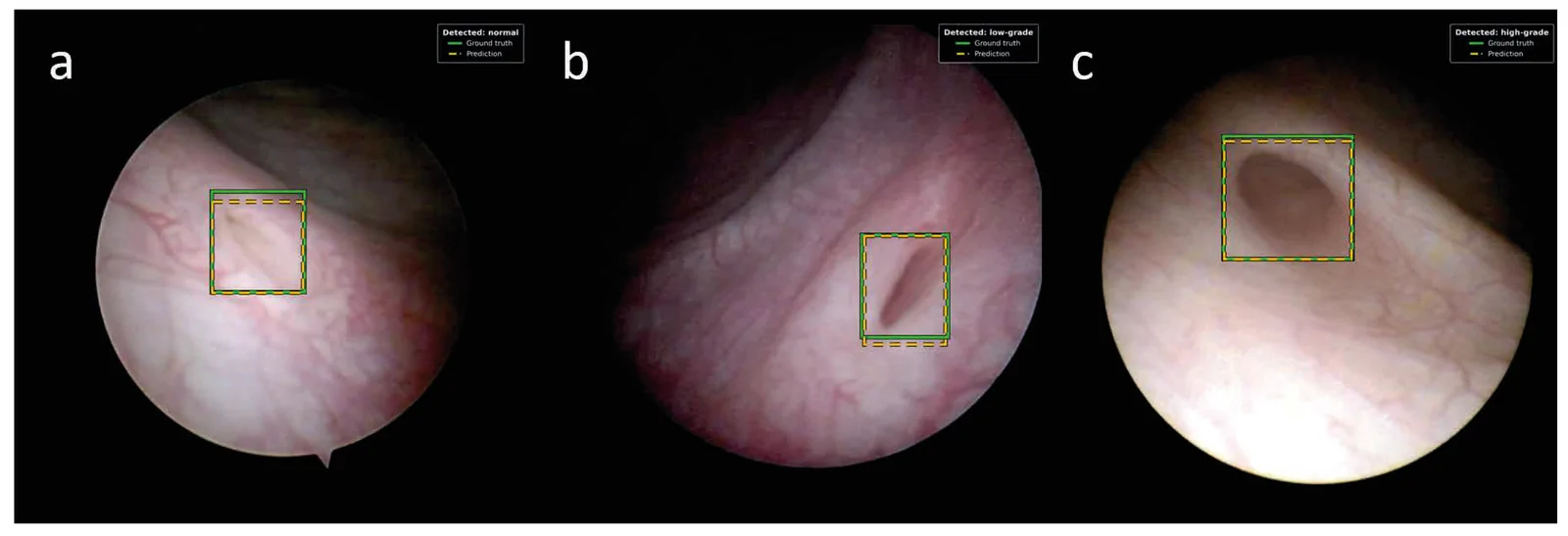
Examples of a ground truth and prediction using YOLOv12n of a normal ostium (**a**), a low-grade (**b**) and a high-grade ostium (**c**).

Training on three-class object detection showed weak results on the test set, as shown in Table 3, with maximum mAP@50 of 0.37 (YOLOv12s) and mAP@50-95 of 0.17 (YOLOv12s). This indicates that the model can only reliably place one out of every 3 target frames correctly, such that it has an overlap of at least 0.5 IoU with the ground-truth label. Similarly, mAP@50-95 of at most 0.17 suggests that the model struggles with highly accurate placement beyond the 0.5 IoU boundary. Examples images are shown in Figure 1.

### 3.2. Task Simplification

Due to these initial weak results, the task was simplified for binary classification of no-/low- and high-grade reflux, based on the same dataset. Binary classification results showed a slight increase in performance yet remained below expectations, achieving a macro average of 0.62 precision, 0.58 recall and 0.49 F1 score. Looking at individual classes, critical cases (high-grade reflux) showed a low recall of 0.26, limiting the clinical utility of this model. Given the simplifications, the model showed only slight improvements, predicting around half the images correctly.

Class-agnostic object detection improved mAP@0.5 to 0.63 (+0.32 mAP@50) from multi-class prediction for YOLOv12n, yielding even larger improvements than the binary classification. In around 2/3 of the frames, the UO could be located correctly, regardless of what class it may entail. This finding suggests that detecting a UO itself is a difficult task, but the model has more difficulty in classifying a UO than finding it.

### 3.3. Sequence Aggregation

Aggregating prediction results over cross-sequence studies substantially improved results. Across longer video sequences, up to 77% of videos reached the correct classification via a majority voting system given a confidence threshold. In addition, in a leave-one-out (LOVO) setting, in which a threshold is not selected optimally but averaged via cross-validation, results remained between 0.69 and 0.77 for all models (Table 4). This suggests that even models that produce subpar results on a per-image basis can reach correct conclusions when deployed over longer sequences of time. In addition, this also allows the suggestion that the reduced performance may not be a fundamental limitation of the model, but rather that individual frames may not carry sufficient information, which averages out across the sequence.

## 4. Discussion

This study represents, to our knowledge, the first application of AI to cystoscopic video analysis for predicting vesicoureteral reflux based on ureteric orifice morphology in children. While frame-based object detection and classification showed poor performance, aggregating predictions across complete cystoscopic sequences substantially improved diagnostic accuracy, reaching up to 77% at the ureteric-orifice level. These findings suggest that diagnostically relevant information is present within cystoscopic examinations but is distributed across multiple frames rather than consistently contained within isolated images.

AI-assisted image analysis has become increasingly integrated into clinical practice, particularly in radiology and gastrointestinal endoscopy, where it supports lesion detection and real-time decision-making [14,15,16,17,18,19]. These successful applications provide a rationale for exploring similar approaches in pediatric cystoscopy.

Recent advances suggest that AI may also support several aspects of VUR management, although current evidence remains limited [20]. Applications of AI in VUR have primarily focused on structured clinical variables or radiological imaging. Most notably, Khondker and colleagues have demonstrated excellent performance for automated grading of reflux on VCUGs, achieving accuracy exceeding 0.8 in both internal and external validation cohorts [21]. The comparatively lower frame-level performance observed in the present study should not be interpreted as evidence that cystoscopic prediction is intrinsically less suitable for AI-based assessment. Rather, it likely reflects the substantially greater complexity of endoscopic image analysis. Unlike previous studies, our model relied exclusively on endoscopic video appearance without incorporating clinical variables or radiological findings. In contrast to standardized radiographic images, cystoscopic videos are characterized by continuous camera motion, variable illumination, fluid-related artefacts, specular reflections, and considerable anatomical variability, all of which increase the difficulty of automated image interpretation and limit model robustness.

Regarding VUR, AI-based models have also been developed to predict the need for VCUG, estimate the risk of recurrent urinary tract infections and predict the endoscopic treatment results [22,23,24]. In addition, large language models have been evaluated as tools for parental counseling [25]. However, grading of VUR on pediatric cystoscopy images and videos using AI has not been performed yet and further studies building on our results need to be performed to evaluate the possibility of AI as a clinical decision-support tool for less experienced practitioners by assisting in the identification of anatomical and pathological features associated with VUR. The ultimate goal could be the implementation of AI-assisted systems that could facilitate earlier and more reliable assessment of patients during cystoscopy which may ultimately complement conventional diagnostic pathways after prospective validation. Furthermore, the integration of VUR detection into the cystoscopic workflow offers the opportunity not only to improve diagnostic efficiency but also to directly guide therapeutic decision-making during the same procedure.

Several factors likely contributed to the limited performance observed on individual frames. Unlike radiological imaging, cystoscopic videos are highly dynamic, with frequent changes in viewing angle, illumination, bladder distension, and partial occlusion of the ureteric orifice by mucosal folds, irrigation fluid, or instrument movement. Specular reflections further reduce image quality and introduce visual artefacts. Moreover, the morphological characteristics associated with reflux are often subtle and may only become apparent when the ureteric orifice is viewed from a favorable angle or during a particular phase of bladder filling. Consequently, many individual frames may contain insufficient diagnostic information for reliable classification, even for experienced pediatric urologists.

The marked improvement observed after sequence-level aggregation supports the concept that cystoscopic diagnosis is inherently a temporal task. During routine cystoscopy, surgeons rarely base their assessment on a single static image but instead integrate information obtained while continuously inspecting the ureteric orifice from multiple perspectives. Majority voting across consecutive frames appears to mimic this clinical decision-making process by reducing the influence of noisy or non-informative images while emphasizing consistently detected morphological features. This observation is consistent with the broader surgical computer vision literature, in which temporal information extracted from video sequences has been shown to improve scene understanding, workflow recognition, and intraoperative decision support compared with approaches based solely on individual frames [26,27].

We opted for the YOLOv12 architecture because it represents a transition toward attention-centric designs in real-time object detection [12]. Cystoscopic video frames present unique challenges, including highly variable lighting conditions, specular reflections and motion blur. YOLOv12 incorporates an attention-based backbone designed to capture global contextual relationships and long-range spatial dependencies more effectively than purely convolutional networks, while still maintaining the low latency required for real-time intraoperative deployment. In the future, however, a systematic benchmark against other model families (e.g., older YOLO generations, different convolutional architectures, etc.) is a logical next step to isolate architectural benefits, alongside the transition to dedicated video-based architectures that model temporal context explicitly.

Our empirical findings indicate that scaling the model capacity tenfold (from YOLOv12n to YOLOv12l) did not yield a corresponding monotonic increase in diagnostic accuracy, with frame-level mAP@50 remaining constrained between 0.31 and 0.37. This non-monotonic variation suggests that the primary bottleneck limiting accuracy is not model capacity or parameter count, but rather the diversity and size of the dataset (specifically, the number of unique ureteric orifices with 117 in total). While the frame count is high (36,604 images), the high visual correlation between consecutive frames from the same sequence means the effective sample size is dictated by the unique anatomical configurations. Future performance improvements will therefore depend heavily on expanding the patient cohort to encompass a broader spectrum of anatomical and clinical variations, rather than merely employing larger or more complex neural network architectures.

Beyond dataset size, the clinical class imbalance significantly influenced the models’ predictive behavior, which represents another key finding. The clinically most critical class (high-grade reflux) was also the least represented, constituting only 19.7% of the included ureteric orifices. Consequently, the models were highly conservative when predicting this class, achieving a high precision of 0.81 but a low recall of 0.26 for high-grade cases. This combination of high precision and low recall is a classic characteristic of deep learning models trained on imbalanced data [28,29]. Notably, this performance asymmetry persisted across both the YOLOv12 detectors and the structurally distinct EfficientNet classifier, indicating that it reflects the underlying composition and inherent difficulty of the endoscopic data rather than the property of a single training configuration. While this low recall is a clear clinical limitation, it highlights the necessity of exploring targeted imbalance remedies in future work (such as class-weighted focal loss or patient-level oversampling) to safely improve diagnostic sensitivity for severe cases.

This study has several limitations. First, it represents a retrospective single-center study with a relatively small number of patients and UOs available for training deep learning models, which also introduced a marked clinical class imbalance that constrained the models’ sensitivity for high-grade cases. Second, although data splitting was performed at the patient level to prevent information leakage, external validation was not available. Third, annotations were based on expert interpretation of cystoscopic morphology, which remains inherently subjective despite standardized labeling procedures. Fourth, only conventional convolutional object detection models were evaluated, whereas dedicated video-based architectures may be better suited for this application. Finally, the heterogeneous indications for cystoscopy among control patients may introduce additional variability in ureteric orifice appearance.

Our results suggest that in the future an intended clinical application could run on the live cystoscopic stream and display an overlay marking the detected ureteric orifice together with the predicted grade and associated confidence. Rather than presenting an isolated prediction for each individual frame, the display would show a continuously updated majority vote over the frames observed so far, which is the intraoperative equivalent of sequence aggregation. Nevertheless, more studies with larger datasets have to be performed to confirm our results and facilitate further development of our model.

In conclusion, this proof-of-concept study demonstrates that AI can extract diagnostically relevant information from pediatric cystoscopic videos for VUR prediction, although frame-level performance remains limited. The substantial improvement achieved through sequence-level aggregation suggests that temporal information is essential for automated interpretation of UO morphology. These findings establish a foundation for future multi-centre studies employing larger datasets including more patients and dedicated video-based deep learning architectures to develop clinically applicable decision-support systems for pediatric endourology.

## Figures and Tables

**Table 1:** Distribution of classes throughout the proposed dataset. Proportions show a class imbalance between no-, low-, and high-grade reflux.

Class	Ureteric Orifices	Proportion
No reflux	68	58.1%
Low-grade (grades II–III)	26	22.2%
High-grade (grades IV–V)	23	19.7%

**Table 2:** Model hyperparameters for training models in the YOLOv12 family. Identical configurations are used across all model sizes besides architecture configuration to ensure fair comparisons of architectures.

Parameter	Value
Hardware	Single NVIDIA RTX 3060, 12GB RAM
Architecture	Per-model definition
Input image size	640 × 640 pixels
Batch size	16 (nominal batch size of 64, gradient accumulation over 4 steps
Epochs	10 (with early stopping, patience of 4)
Optimizer	AdamW
Initial learning rate	1.43 × 10^−3^
Learning rate schedule	Linear decay to 1% of the initial value (final 1.56 × 10^−4^) with a 3-epoch warm-up
Momentum	0.9 (warm-up momentum 0.8)
Weight decay	5 × 10^−4^
Loss weights	Box 7.5, classification 0.5, distribution focal loss 1.5
Augmentation	Mosaic, horizontal flip (*p* = 0.5), HSV jitter (0.015, 0.7, 0.4), translation 0.1, scaling 0.5
NMS	IoU threshold 0.7, maximum 300 detections per image
Numerical precision	Automatic mixed precision
Seed	0, deterministic mode enabled

**Table 3:** Three-class object detection results for models from the YOLOv12 family. The metrics mAP@50, mAP@50-95, and accuracy are reported.

Model	mAP@50	mAP@50-95	Per-Frame Accuracy
YOLOv12n	0.31	0.14	0.40
YOLOv12s	0.37	0.17	0.42
YOLOv12m	0.31	0.14	0.36
YOLOv12l	0.32	0.14	0.34

**Table 4:** Sequence aggregation results from models of the YOLOv12 family. Per-UO accuracy for an optimal threshold, per-UO accuracy for a threshold determined via leave-one-out cross-validation, as well as the LOVO 95%-confidence interval are reported.

Model	Per-UO Accuracy (Static)	Per-UO Accuracy (LOVO)	95%-CI (LOVO)
YOLOv12n	0.74 (@0.05)	0.71	[0.57–0.86]
YOLOv12s	0.74 (@0.15)	0.69	[0.51–0.83]
YOLOv12m	0.74 (@0.05)	0.71	[0.57–0.86]
YOLOv12l	0.77 (@0.00)	0.77	[0.63–0.89]

## Data Availability

The data supporting the reported results are not publicly available due to institutional data governance restrictions. Requests for data access may be directed to the corresponding author.

## Abbreviations

The following abbreviations are used in this manuscript:

VUR	vesicoureteral reflux
UO	ureteric orifice
VCUG	voiding cystourethrography
AI	artificial intelligence
UTI	urinary tract infection
ML	machine learning
UPJO	ureteropelvic junction obstruction
CVAT	computer vision annotation tool
YOLO	you only look once
LOVO	leave-one-out
